# Activin and TGFβ use diverging mitogenic signaling in advanced colon cancer

**DOI:** 10.1186/s12943-015-0456-4

**Published:** 2015-10-24

**Authors:** Jessica Bauer, Ozkan Ozden, Naomi Akagi, Timothy Carroll, Daniel R. Principe, Jonas J. Staudacher, Martina E. Spehlmann, Lars Eckmann, Paul J. Grippo, Barbara Jung

**Affiliations:** Department of Medicine, Division of Gastroenterology and Hepatology, University of Illinois at Chicago, 840 South Wood Street, 738A CSB, Chicago, IL 60612 USA; Department of Internal Medicine III, Cardiology and Angiology, University Hospital Schleswig-Holstein, Kiel, Germany; Department of Medicine, University of California, San Diego, CA USA

**Keywords:** Colon cancer, Activin, TGFβ, PI3K, Mitogenic signaling, p21

## Abstract

**Background:**

Understanding cell signaling pathways that contribute to metastatic colon cancer is critical to risk stratification in the era of personalized therapeutics. Here, we dissect the unique involvement of mitogenic pathways in a TGFβ or activin-induced metastatic phenotype of colon cancer.

**Method:**

Mitogenic signaling/growth factor receptor status and p21 localization were correlated in primary colon cancers and intestinal tumors from either AOM/DSS treated ACVR2A (activin receptor 2) −/− or wild type mice. Colon cancer cell lines (+/− SMAD4) were interrogated for ligand-induced PI3K and MEK/ERK pathway activation and downstream protein/phospho-isoform expression/association after knockdown and pharmacologic inhibition of pathway members. EMT was assessed using epithelial/mesenchymal markers and migration assays.

**Results:**

In primary colon cancers, loss of nuclear p21 correlated with upstream activation of activin/PI3K while nuclear p21 expression was associated with TGFβ/MEK/ERK pathway activation. Activin, but not TGFβ, led to PI3K activation via interaction of ACVR1B and p85 independent of SMAD4, resulting in p21 downregulation. In contrast, TGFβ increased p21 via MEK/ERK pathway through a SMAD4-dependent mechanism. While activin induced EMT via PI3K, TGFβ induced EMT via MEK/ERK activation. *In vivo,* loss of ACVR2A resulted in loss of pAkt, consistent with activin-dependent PI3K signaling.

**Conclusion:**

Although activin and TGFβ share growth suppressive SMAD signaling in colon cancer, they diverge in their SMAD4-independent pro-migratory signaling utilizing distinct mitogenic signaling pathways that affect EMT. p21 localization in colon cancer may determine a dominant activin versus TGFβ ligand signaling phenotype warranting further validation as a therapeutic biomarker prior to targeting TGFβ family receptors.

**Electronic supplementary material:**

The online version of this article (doi:10.1186/s12943-015-0456-4) contains supplementary material, which is available to authorized users.

## Introduction

Colon cancer remains the second deadliest cancer in the United States with an estimated 136,830 new cases and 50,310 deaths in 2014 [1]. The overall incidence, as well as cancer-related mortality, have both decreased over the past 10 years, which has been attributed to enhanced screening and early detection. However, once colon cancer has metastasized, the five year survival remains poor [1]. Further, and disturbingly, the number of young patients with metastatic disease is increasing [1]. Understanding the switch to metastatic behavior and developing therapeutic strategies to target metastatic signaling are key unmet clinical challenges. This understanding will lead to the generation of functional biomarkers to better predict patient risk and potential treatment response to individual pathway inhibition.

Recent efforts in cancer genome comprehensive sequencing have confirmed key genes whose mutations can drive tumorigenesis [2] and have solidified components of the Transforming Growth Factor (TGF) β superfamily as drivers of pathogenesis in colon cancer. These include inactivating mutations in the TGFβII receptor (TGFBR2), the activin receptor 2A (ACVR2A) and downstream signaling molecule SMAD4 [2]. TGFβ and activin are involved in the regulation of cell proliferation, differentiation, migration and apoptosis [3–5]. Activin and TGFβ utilize a specific type I/type II receptor complex for signal transduction [6–8]. In the canonical pathway, ligand binding leads to activation of SMAD2/3/4 proteins, translocation to the nucleus and transcriptional regulation of target genes to affect growth suppression and p21 upregulation. The non-canonical signaling pathway is SMAD4-independent and may engage other signaling pathways [4, 5]. Activin and TGFβ both have dual and opposing roles in colon carcinogenesis as they may promote growth suppression, as well as migration and metastasis in more advanced colon cancer, also known as the molecular switch [9–12]. TGFβ itself has opposing functions: in early stage colon cancer, the TGFβ super family is growth suppressive, while in advanced disease, high TGFβ serum and stroma levels are associated with poor prognosis [13, 14]. Similarly, high levels of plasma activin in pancreatic cancer patients are significantly associated with decreased overall survival and increased distant metastases [15]. Therefore, it is critical to understand the switch from growth suppression to proliferation for TGFβ and activin signaling. In order to identify targets which may directly affect metastatic behavior, we need to understand how the respective pathways intersect with other signaling cascades and how specifically, activin and TGFβ differ in their effects.

We have previously shown that in colon cancer cells, the cyclin dependent kinase (CDK) inhibitor p21 is a downstream target of both activin and TGFβ [9]. In primary colon cancer tumors, we observed that nuclear p21 localization correlated with TGFBR2 expression while loss of nuclear p21 is associated with ACVR2A expression, respectively [9]. To understand the contribution of each signaling pathway to the net signaling measured in tumor samples, we further dissected activin and TGFβ signaling in colon cancer cells. Despite shared SMAD2/3/4 signaling of activin and TGFβ, we observed opposing downstream effects on p21; namely TGFβ induces SMAD-dependent upregulation of p21, whereas activin leads to SMAD-independent downregulation of p21 via increased proteasomal degradation [9]. Additionally, since SMAD4 by itself is frequently mutated and inactivated in colon cancer [16], it is important to understand the SMAD-independent signaling of activin and TGFβ and the downstream impact on metastatic processes.

While TGFβ induced EMT in cancer has been studied in detail, there are only a few conflicting reports on activin and EMT [17, 18]. TGFβ has been shown to induce an invasive phenotype and EMT [17, 19, 20], but the precise mechanism is not well described. Activin’s effects on EMT have not been studied in detail to date. Reports in the literature indicate that increased expression of pAKT is associated with the loss of p21 expression in adenoid cystic carcinomas [21] while ligand activation of TGFBR2 can activate the MEK/ERK pathway [5]. In colon cancer, the influence of mitogenic signaling on the regulation of EMT and the effect on a metastatic phenotype in colon cancer through TGFβ is not well known. However, previous reports have implicated PI3K signaling in EMT following TNF ligand stimulation in colon cells [22]. Phosphatidylinositol-3’-kinase (PI3K) signaling is involved in the regulation of several key cellular processes such as cell growth, survival, motility and proliferation which are involved in tumorigenesis [23]. The PI3K pathway plays a prominent role in many cancers; in colon cancer specifically, upstream activation or gain of function mutations are common [24]. Akt, one of the downstream effectors of PI3K signaling, regulates apoptosis and cell cycle progression. Activation of the PI3K/Akt pathway can arise through various mechanisms and is associated with a poor prognosis in a number of cancers [24]. MEK/ERK mitogenic signaling is also commonly activated in tumors and affects regulation of the cell cycle as well as senescence [25]. ERK may directly influence p21 localization and stability [25], but the effects of TGFβ/activin–mediated mitogenic signaling have not been studied.

In order to delineate the respective and unique contributions of activin and TGFβ in anticipation of individual pathway inhibition in metastatic signaling, we first interrogated the association of PI3K/Akt and MEK/ERK signaling with the status of activin and TGFβ receptor expression in colon cancer patients. Then, we further dissected downstream use of mitogenic signaling pathways in activin and TGFβ-specific signaling as well as effects on p21 regulation, EMT, and migration in colon cancer. Better understanding of the distinct effects of activin and TGFβ signaling in metastatic disease is crucial in anticipation of specific pathway inhibition via small molecules. Moreover, we suggest nuclear p21 as a potential therapeutic biomarker that may accurately distinguish whether activin or TGFβ signaling is dominant in a given colon cancer patient. This study adds to the growing complexity of preclinical information necessary to plan much needed trials in advanced colon cancer disease using pathology-guided therapeutics.

## Results

### Loss of nuclear p21 is indicative of activin/pAkt activation, while nuclear p21 is associated with TGFβ/pERK activation in primary colon cancer tissues

**Fig. 1:**
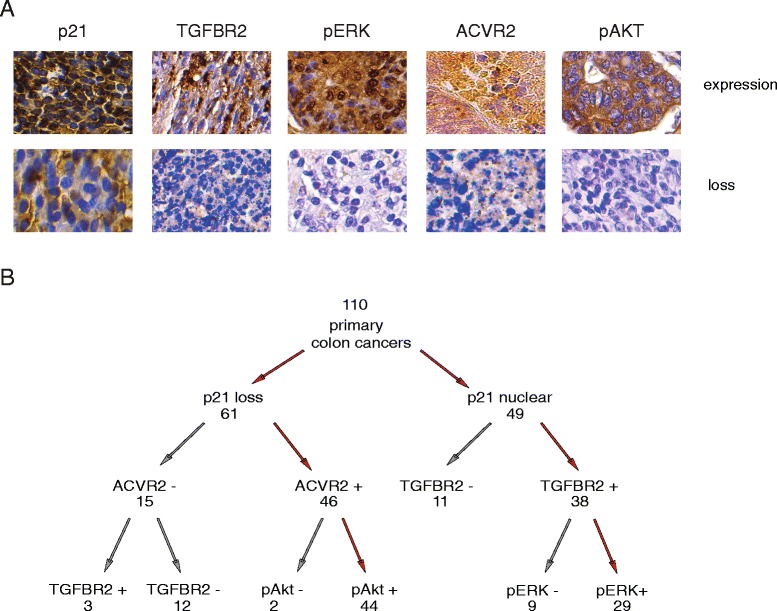
Loss of nuclear p21 is indicative of activin/pAkt activation, while nuclear p21 is associated with TGFβ/pERK activation in primary colon cancer tissues. In human colon cancer tissue, loss of nuclear p21 is associated with ACVR2A/pAkt expression while nuclear p21 is associated with TGFBR2/pERK expression. A total of 110 primary human colon cancer tissues were stained for ACVR2A/TGFBR2/p21/pERK/pAkt expression and pathway expression associations were determined. **a** Representative colon cancer tissues showing nuclear (p21/pERK) or cytosolic (TGFBR2/ACVR2A/pAkt) expression (upper panel) versus loss (lower panel). **b** Schematic of colon cancer signaling pathways based on signal component staining. Dominant pathways with p21 expression as diverging point drawn in red (p21 loss/ACVR2A+/pAkt + and p21 expression/TGFBR2+/pERK+, respectively). For statistical analysis, see Table 1

We have previously observed that loss of nuclear p21 expression correlates with either presence of ACVR2A or absence of TGFBR2 in primary colon cancers [9]. Here we have expanded those observations through assessing the relative expression of ACVR2A/TGFBR2 receptors compared to paired normal tissue through immunohistochemistry in 110 primary colon cancer tumors and examined the impact of receptor status on p21 localization and activation of pAkt and pERK mitogenic signaling pathways (Fig. 1 and Table 1). Increased nuclear p21 was associated with TGFBR2 expression (Additional file 1: Table S2) and TGFBR2 expression correlated with an increase in pERK (*p* = 0.0019, Table 1A) which is consistent with a TGFβ/pERK/nuclear p21 axis. Loss of nuclear p21 was associated with ACVR2A expression (Additional file 1: Table S2) and an increase in pAkt expression (*p* = 0.001, Table 1B). In line with our hypothesis, pERK and pAkt expression were not associated with each other (*p* = 1). This suggests that nuclear p21 expression may be used as an indicator of net upstream TGFβ or activin mitogenic signaling, or more functionally, TGFβ/SMAD growth suppressive, versus activin/migratory signaling, which would aid in planning inhibition with small molecules. Statistical analysis of nuclear p21 versus TGFBR2 expression and loss of nuclear p21 versus ACVR2A expression showed no significant differences. While there was a trend towards correlation of ACVR2A expression and loss of p21 in advanced stages the relatively small sample size and predominance of stage III cases limited this analysis. To further understand the distinct individual regulation of activin and TGFβ in colon cancer, we next dissected pathway components in colon cancers cells *in vitro*.

**Table 1:** Correlation of signaling pathway expression in primary colon cancer slides

A) pERK and TGFBR2 are correlated with a *p* = 0.0019 (Fisher’s exact test)				
	TGFBR2+	TGFBR2-	total	*p* = 0.0019
pERK +	41	22	63	
pERK -	16	31	47	
total	57	53	110	
B) pAKT and ACVR2 are correlated with a *p* = 0.0001 (Fisher’s exact test)				
	ACVR2+	ACVR2-	total	*p* = 0.0001
pAkt +	44	16	60	
pAkt -	18	32	50	
total	62	48	110	

### Activin, but not TGFβ, utilizes PI3K/Akt to downregulate p21 in a SMAD4-independent manner

We previously demonstrated that activin downregulates p21 independently of SMAD4 [9], a key downstream signaling component of both the activin and TGFβ pathways. As PI3K/Akt signaling is a known regulator of p21 in cancer [26], to elucidate the SMAD4-independent signaling of activin in colon cancer, we hypothesized that the activin receptor directly engages the PI3K/Akt pathway upstream of SMAD4. To test this hypothesis, we assessed whether activin and/or TGFβ led to PI3K activation via primary receptor association with the p85 regulatory subunit of PI3K and the subsequent downstream increase in phosphorylation of Akt in *SMAD4* wild type and null colon cancer cell lines.

**Fig. 2:**
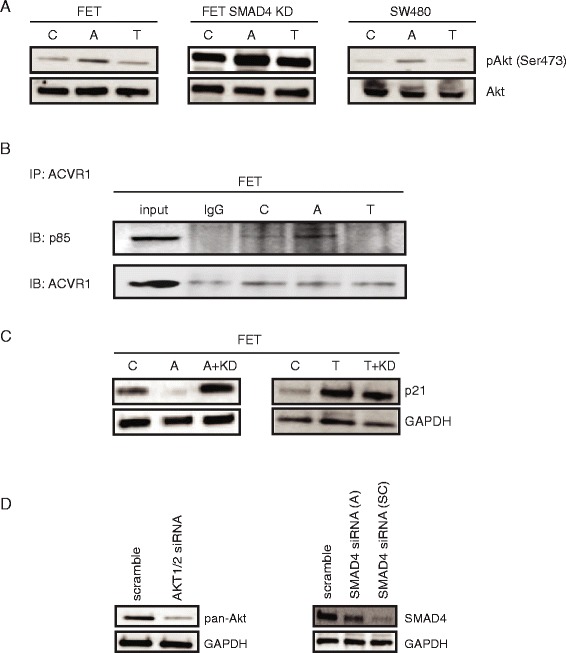
Activin but not TGFβ utilizes PI3K/Akt to downregulate p21 in a SMAD4-independent manner. **a** Activin but not TGFβ leads to PI3K activation in a SMAD4-independent manner. *ACVR2A/TGFBR2* wild type FET cells, FET with *SMAD4* knockdown, and the *SMAD4*-null colon cancer cell line SW480 were stimulated with activin or TGFβ for 24 h following serum starvation. pAkt level was determined by Western Blot. Akt was used as a loading control. pAkt increased after cells were treated with activin but not TGFβ. **b** ACVR1B, ACVR2A’s primary binding partner, interacts with p85, the regulatory subunit of PI3K in an activin-dependent manner. Co-immunoprecipitation (Co-IP) with *ACVR2A/TGFBR2* wild type FET cells were used to detect a protein-protein interaction between ACVR1B and p85. **c** p21 downregulation is dependent on Akt, a PI3K downstream target. *ACVR2A/TGFBR2* wild type FET cells were transfected with siRNA to Akt1/2 (KD) and treated with activin or TGFβ for 24 h following serum starvation. Activin-induced downregulation of p21 was abrogated after Akt1/2 knockdown implicating Akt in activin-induced p21 regulation. **d** Knock down of downstream target in FET cell. *ACVR2A/TGFBR2* wild type FET cells were transfected with siRNA Akt1/2 and siRNA SMAD4. Resulting loss of respective protein expression is shown using Western blotting. For siRNA SMAD4 we tested two different siRNA from Ambion (A: middle panel) and Santa Cruz (SC: right panel) and the latter was used in all our experiments. (C control; A Activin; T TGFβ; KD siRNA Akt1/2; IP immunoprecipitation)

Using *ACVR2A/TGFBR2* wild type FET colon cancer cells, FET with *SMAD4* knockdown, and the *SMAD4*-null colon cancer cell line SW480, we found a SMAD4-independent increase in pAkt Ser473 after activin treatment compared to control and TGFβ treatment (Fig. 2a). Conversely, pAkt Thr308 did not change (data not shown) consistent with phosphorylation through the mTOR/rictor pathway and not the ER stress pathway [27].

While ACVR1B and p85 co-localized following activin treatment in *ACVR2A/TGFBR2* wild type FET cells (Fig. 2b), TGFβ treatment did not increase basal levels of p85/TGFBR1 interaction (Fig. 2b). These data were confirmed in FET *SMAD4* knock down cells and SW480 cells (data not shown), indicating a SMAD4-independent process. To assess the interaction of p85 with other activin/TGFβ receptor isoforms, we co-immunoprecipitated p85 with ACVR2A, TGFBR1, and TGFBR2 and determined that ACVR1B has the highest affinity for p85. The interaction between ACVR2A and p85 was less than the interaction between ACVR1B and p85. (Additional file 2: Figure S1), and there was no interaction between TGFBR1 or TGFBR2 and p85 (Additional file 2: Figure S1). Therefore, we conclude that stimulation by activin ligand leads to a relative dominance of the interaction of p85 with ACVR1B (which is specific for activin signaling) over the less specific ACVR2A.

To determine if the activin-stimulated interaction of ACVR1B and p85 results in activation of the PI3K pathway, we knocked down the two most common Akt isoforms (Akt1/2) and assessed p21 expression. In the FET cells, the activin-induced downregulation of p21 was abrogated following Akt1/2 knockdown (Fig. 2c), however, Akt1/2 knockdown had no effect on p21 expression after TGFβ treatment. This implies that activin-induced downregulation of p21 involves ACVR1B interaction with p85 to activate PI3K/Akt signaling. In contrast, TGFβ-mediated upregulation of p21 does not utilize PI3K signaling.

### TGFβ, but not activin, stabilizes p21 via SMAD4 and MEK/ERK

**Fig. 3:**
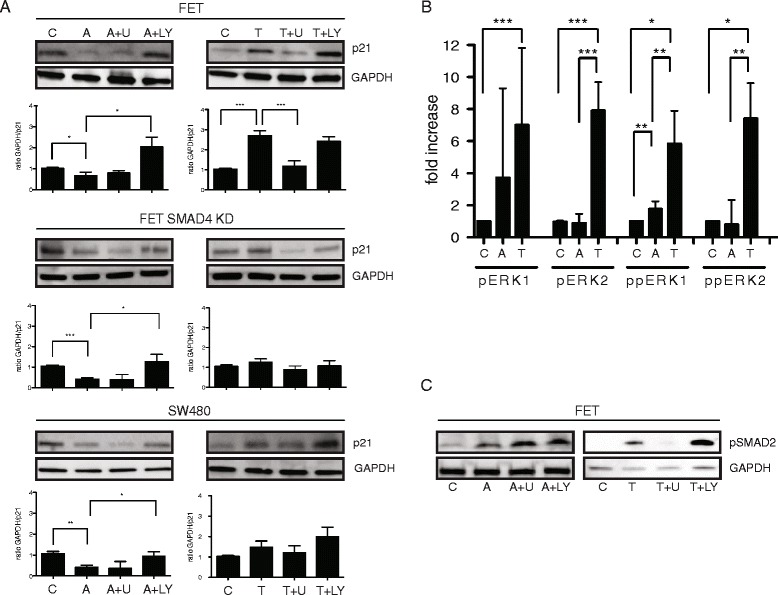
TGFβ but not activin stabilizes p21 via SMAD4 and MEK/ERK. **a** TGFβ-induced p21 upregulation is MEK/ERK dependent and not influenced by PI3K inhibition. In contrast, activin-induced p21 downregulation is dependent on PI3K signaling, but not dependent on SMAD4. *ACVR2A/TGFBR2* wild type FET cells, FET with *SMAD4* knockdown, and the *SMAD4*-null colon cancer cell line SW480 were treated with activin or TGFβ for 24 h 30 min after pharmacologic inhibition of PI3K (LY 290042) or MEK1/2 inhibition (U0126) and p21 levels determined by Western blot. GAPDH was used as a loading control. Three independent experiments entered the densitometric analysis shown below the representative blots (*p < 0.05, **p < 0.01, ***p < 0.001). For simplicity, we only show level of significance between control versus activin; control versus TGFβ; and activin versus LY and TGFβ versus U. **b** TGFβ treatment prominently increases the phosphorylation of ERK1/2 in colon cancer cells. Isoelectric point immunoassay was performed in FET cells treated with activin or TGFβ and pERK1/2 and ppERK1/2 compared to vehicle control. Five independent experiments were performed (*p < 0.05, **p < 0.01, ***p < 0.001). **c** TGFβ utilizes MEK/ERK to induce pSMAD2. *ACVR2A/TGFBR2* wild type FET cells were treated with activin, TGFβ or vehicle control for 24 h following pretreatment with PI3K and MEK/ERK inhibition. pSMAD2 was determined by Western blot analysis and GAPDH was used as a loading control. Activin-induced pSMAD2 increase is independent from PI3K and MEK/ERK, but inhibition of MEK/ERK abolishes TGFβ-induced upregulation of pSMAD2 (A + LY Activin + LY; A + U Activin + U0126; T + LY TGFβ + LY; T + U TGFβ + U0126)

To further dissect the relevant downstream pathways of TGFβ and activin-induced regulation of p21, we utilized FET, FET with *SMAD4* knockdown, and *SMAD4*-null SW480 and treated with pharmacologic inhibitors of PI3K (LY 290042) or MEK (U0126) prior to ligand treatment. Expression of p21 was then measured by immunoblotting. Inhibition of PI3K blocked the activin-induced downregulation of p21 expression regardless of SMAD4 status (Fig. 3a, left panels), whereas TGFβ induction of p21 was dependent on both MEK/ERK and SMAD4 (Fig. 3a, right panels). These results indicate that TGFβ-induced upregulation of p21 engages the MEK/ERK pathway, while activin-induced p21 downregulation is unaffected by MEK/ERK. To simultaneously quantify the impact of ligand treatment (TGFβ or activin versus untreated control) on pERK1/2 and ppERK1/2 isoforms, we employed isoelectric point immunoassay analysis. We observed an increase of pERK1/2 and ppERK1/2 isoforms after TGFβ treatment compared to both control and activin treatment (Fig. 3b) confirming that TGFβ, but not activin, induces the MEK/ERK signaling. Further, we found that pSMAD2 induction following TGFβ was dependent on MEK/ERK (Fig. 3c). The inhibitors alone do not have an effect on p21 and pSMAD2 expression (data not shown). Therefore, in colon cancer cells, TGFβ stabilizes p21 via MEK/ERK in a SMAD4-dependent manner resulting in upregulation of p21.

### Activin and TGFβ use distinct mitogenic signaling to affect SMAD4- independent migration

**Fig. 4:**
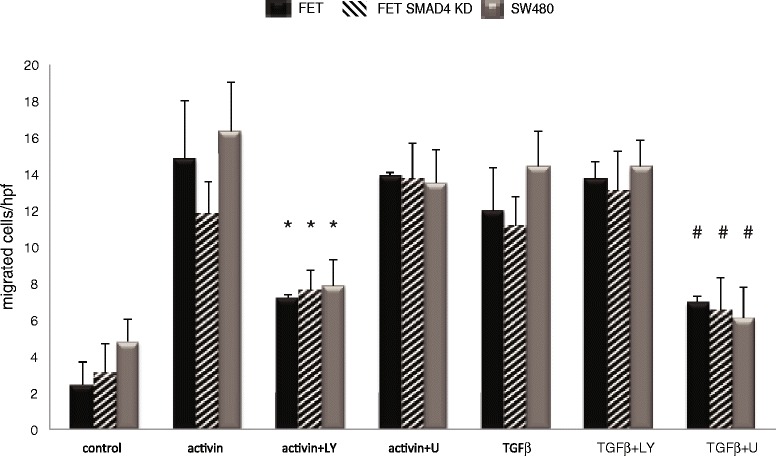
Activin and TGFβ use distinct mitogenic signaling to affect SMAD4- independent migration. Activin and TGFβ are both inducers of migration independent of SMAD4. *ACVR2A/TGFBR2* wild type FET cells, FET with *SMAD4* knockdown, and the *SMAD4*-null colon cancer cell line SW480 were seeded in transwell plates, serum starved, and pretreated with pharmacological inhibitors of PI3K (LY 290042) or MEK (U0126). Activin and TGFβ-induced migration is SMAD4-independent. Activin-induced migration is decreased in the absence of PI3K signaling and TGFβ-induced migration is MEK/ERK dependent. Inserts show representative fields with migrated FET cells. Graph shows data from 4 independently performed experiments (*p < 0.05 Activin versus Activin + LY; #p < 0.05 TGFβ versus TGFβ + U0126)

Activin and TGFβ are both inducers of migration [9, 10] and mitogenic signaling is associated with a migratory phenotype [24]. To determine if TGFβ- and activin-induced migration involves MEK/ERK or PI3K signaling respectively, we inhibited PI3K or MEK in our colon cancer cell models and assessed the impact on migration following ligand stimulation. Activin-induced migration decreased significantly following inhibition of PI3K irrespective of SMAD4 status and was not affected by MEK inhibitors. However, TGFβ-induced migration was significantly decreased after MEK inhibition, independently of SMAD4 expression, and was not affected by PI3K inhibition (Fig. 4, Additional file 3: Figure S2). These data suggest that the migration induced by TGFβ and activin is independent of SMAD4 and employs distinct mitogenic signaling.

### Activin and TGFβ induce epithelial to mesenchymal transition via distinct mitogenic signaling pathways

**Fig. 5:**
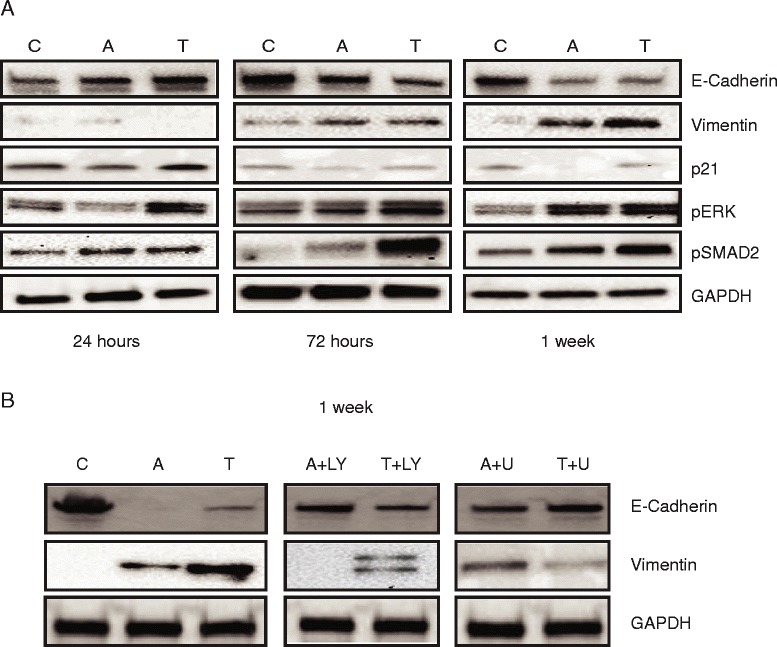
Activin and TGFβ induce epithelial to mesenchymal transition via distinct mitogenic signaling and downregulation of p21. **a** Both TGFβ and activin treatment induce a decrease of E-Cadherin and an increase in vimentin, indicative of EMT while p21 is downregulated. TGFβ-induced p21 upregulation normalizes over time while activin induced downregulation of p21 persists. *ACVR2AsTGFBR2* wild type FET colon cancer cells were treated with activin or TGFβ and lysed after 24 h, 72 h or 1 week, respectively. EMT was determined using E-Cadherin as an epithelial and vimentin as a mesenchymal marker. p21, pERK, pSMAD2 during activin and TGFβ-induced EMT were also interrogated. Phosphorylation of SMAD2 and ERK increased over time after TGFβ treatment. **b** Inhibition of PI3K following activin and inhibition of MEK following TGFβ treatment leads to a decrease in EMT. FET cells were pretreated with PI3K (LY 290042) or MEK (U0126) prior to stimulation with activin or TGFβ and lysed after one week. EMT was determined using E-Cadherin as an epithelial marker. Representative blots of 3 independent experiments are shown

Consistent with a role in oncogenic signaling, TGFβ has been shown to induce epithelial to mesenchymal transition (EMT) in advanced cancer [28]. However, activin’s effects on EMT are currently unclear. As activin downregulates p21 and accordingly opposes EMT in breast cancer [29], we assessed the potential contribution of activin and its downstream signaling to EMT in colon cancer. For this, FET cells were treated with either activin or TGFβ, and expression of E-Cadherin (epithelial phenotype) and vimentin (mesenchymal phenotype) were determined after 24 h, 72 h and 1 week. Seventy-two hours and 1 week after TGFβ or activin treatment, E-Cadherin expression was reduced compared to controls while vimentin expression was increased (Fig. 5a), consistent with EMT. Treatment with TGFβ led to an increase in p21 at 24 h, which was not sustained at longer time points. However, the activin-induced downregulation of p21 was stable over time (Fig. 5a). This is consistent with our previous data suggesting that activin may be a more potent inducer of migration [9], as in cells with intact SMAD4, TGFβ induced p21 may counteract migration [9] and EMT at early time points.

As we observed that pERK plays a role in phosphorylation of SMAD2 (Fig. 3), we next assessed the role of MEK/pSMAD2 or PI3K in TGFβ or activin-mediated EMT. Phosphorylation of ERK and SMAD2 increased after TGFβ treatment relative to control at all the time points examined (Fig. 5a). Further, concomitant inhibition of PI3K led to a downregulation of activin-induced EMT while inhibition of MEK led to a decrease of EMT after TGFβ treatment evident through changes in E-Cadherin and vimentin expression (Fig. 5b). Taken together, despite their shared regulation of SMAD signaling, activin and TGFβ diverge in their downstream signaling to affect induction of an invasive phenotype in colon cancer.

### Loss of ACVR2A *in vivo* is associated with pAkt down regulation in intestinal tumors

**Fig. 6:**
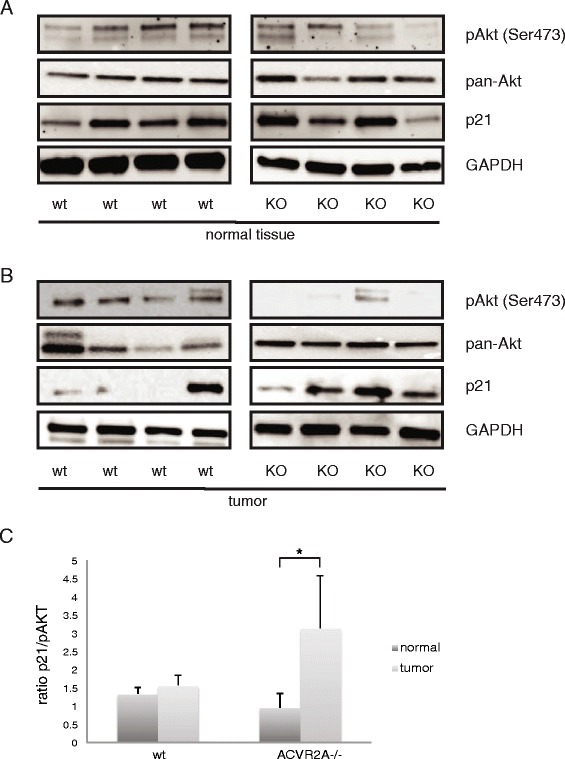
Loss of ACVR2A *in vivo* is associated with pAkt downregulation in intestinal tumors. Loss of ACVR2A leads to pAkt downregulation and p21 upregulation in *ACVR2A* knockout KO mice. *ACVR2A* wild type (wt) and *ACVR2A* KO mice were used in a DSS/AOM intestinal cancer model. Mice were injected intraperitoneally with AOM and after 5 days were given 3 cycles of DSS. Lysates from normal (**a**) and 10.1186/s12943-015-0456-4 intestinal tumor tissue (**b**) were probed for p21, pan-Akt, and pAkt expression via Western blot. Tumor and normal tissue from four different mice from each group of *ACVR2A* wt and *ACVR2A* KO mice is shown. **c** Loss of *ACVR2A* is associated with a decrease in pAkt and an increase of p21, which is most pronounced in intestinal tumor tissue. Immunobloy signal was quantified by densitometry and the ratio of p21 to pAKT expression was determined

To explore the mechanisms through which activin affects PI3K signaling *in vivo*, *ACVR2A* knockout mice (KO) and *ACVR2A* control wild type (wt) mice were used in a murine model of colitis-associated colorectal cancer. While there was a trend towards more intestinal cancers in the challenged *ACVR2A* knock outs compared to wild type mice, this was not statistically significant, underscoring the likely overlap/synergy of TGFβ family member signaling in colon cancer (Fig. 6). We then isolated tumor tissues and paired normal tissues from the colons of study animals to quantify pAkt (Ser 473), pan Akt and p21 expression. The level of pan Akt was similar across mice (Fig. 6). However, in tumor tissue of *ACVR2A* KO mice, we observed a decrease in pAkt and an increase in p21 compared to normal tissue (Fig. 6b right panel) consistent with an activin/PI3K/Akt involvement in p21 downregulation in colon cancer. An increase of p21 and a decrease in pAKT in the tumor tissue of ACVR2A KO mice is significant (Fig. 6c).

**Fig. 7:**
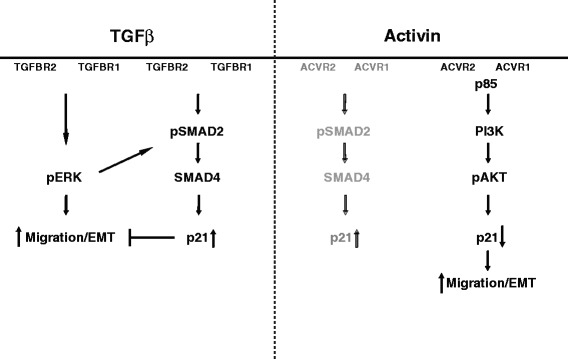
Parallel activin and TGFβ signaling in advanced colon cancer. TGFβ and activin both share SMAD4 signaling to upregulate p21. However, in colon cancer, there is ligand-specific SMAD4-independent signaling utilizing distinct mitogenic signaling. Moreover, activin dominantly induces downregulation of p21 via PI3K/Akt signaling over early SMAD4-dependent p21 upregulation in colon cancer (non-dominant pathway indicated in grey). Net nuclear p21 expression in colon cancer may be a functional surrogate of intact TGFβ/SMAD growth suppression and a negative possible predictor of growth enhancing response to TGFβ pathway inhibition. In contrast, colon cancers with loss of nuclear p21 may benefit from activin, TGFβ or combination inhibitory therapy. In summary, there is complex parallel signaling with feedback loops operative in colon cancer downstream of activin and TGFβ. In order to predict net functional effects of targeted pathway disruption on tumor behavior, it is crucial that the interplay of pathways is fully appreciated to minimize unwanted side effects

In summary, we show differential and parallel activin and TGFβ signaling in colon cancer. These pathways involve both SMAD4-dependent and independent signaling cascades and utilize distinct mitogenic signaling effectors (Fig. 7). Both TGFβ and activin may signal via SMADs to upregulate p21 at early time points [9]. In colon cancer, this pathway is non-dominant [9] and activin preferentially downregulates p21 via PI3K/Akt in a SMAD4-independent fashion. This is associated with an increase in metastatic phenotype as evidenced by increased migration and EMT. In addition, loss of nuclear p21 correlates with activin/PI3K signaling activation in primary colon cancers, however, some cancers retain net nuclear p21, suggestive of dominant growth suppressive TGFβ/SMAD signaling. These data suggest further validation of nuclear p21 as a therapeutic biomarker, and indicate caution should be exercised when inhibiting TGFβ signaling in colon cancers with retained nuclear p21.

## Discussion

TGFBR2 and ACVR2A are commonly mutated in microsatellite unstable (MSI) colon cancers, which is the second most common genomic subtype [30]. The characteristic defects in mismatch repair result in mutations in repetitive sequences termed microsatellites [31–34]. Both TGFBR2 and ACVR2A harbor coding microsatellites and mutations in MSI colon cancers which are associated with loss of protein expression [35]. In addition, mutations in non-MSI cancers may occur in their common downstream signaling component SMAD4 [3]. While all this underscores that the TGFβ superfamily undoubtedly is important in colon cancer, the respective contribution of each pathway is still poorly understood. Detailing such contributions and functional net effects in colon cancer is crucial when envisioning treatment with the now emerging TGFβ pathway inhibitors.

It is conceivable that the redundancy in downstream SMAD signaling as exhibited by activin and TGFβ may lead to pathway rescue [36]; however, given the complex cross talk between TGFβ family members and other signaling pathways, this is likely an oversimplification. We and others have found SMAD-independent signaling is associated with distinct functional effects that occur parallel to SMAD signaling in *SMAD4* wild type colon cancer cells [9]. In addition, when both signaling cascades are intact, there is downstream divergence in the regulation of p21 [9], which may remain dominant in some cancers and underscores the complexity of pathway interactions.

It is clear that TGFβ family signaling is modified dependent on cellular context [3]. Parallel signaling of TGFβ and activin likely both play a role in the development of advanced colon cancer, and net signaling will be affected by selective pathway abrogation as a result of the mutational heterogeneity of each tumor. Understanding this net signaling effect is crucial to the development of therapeutic approaches which target the pro-migratory function in advanced colon cancer while protecting the anti-proliferative functions of TGFβ in early colorectal cancer development. In this report, we have dissected activin and TGFβ signaling divergence and their net functional effects with the aim of identifying a potential therapeutic marker.

Colon cancer treatment is entering the realm of precision medicine. Metastatic colon cancers are routinely tested for mutant *KRAS* to determine suitability for biologic adjuvant therapy with the anti-epidermal growth factor receptor (EGFR) antibody, cetuximab [37], as surprisingly, patients with mutant *KRAS* fared worse with anti-EGFR therapy than without treatment [37]. Small molecule inhibitors for both TGFBR1/2 and ACVR1B are available and conceivably could be used as adjuvant therapies in advanced colon cancer, but biomarkers to determine treatment suitability and specifically to avoid augmentation of oncogenic signaling are lacking. This need is underscored by a recent report that in pancreatic cancer inactivation of PI3K/mTOR signaling led to compensatory increase in mitogenic MEK/ERK signaling [38]. Here, we have dissected signaling overlap and divergence of activin and TGFβ signaling as well as respective mitogenic signaling and we now suggest nuclear p21 expression as a potential read out for active upstream growth suppressive TGFβ/SMAD signaling. Further, we caution that inhibition of TGFβ in such cancers may lead to increased growth.

Mitogenic signaling pathways are frequently activated in cancers and the subject of intense investigation for therapeutic intervention. The MAPK pathway is commonly activated in tumors by direct mutation or overexpression of upstream molecules such as BCR-ABL and EGFR. TGFβ can activate MAPK and PI3K dependent on cell type and culture condition [3, 39]. We now observe that in colon cancer cells MAPK is activated by TGFβ by a SMAD-dependent mechanism leading to regulation of p21 expression.

In contrast, we also report that PI3K is an active signaling component of activin/SMAD-independent mediated downregulation of p21 and associated with a metastatic phenotype. Consistent with an activin/PI3K/pAkt–induced p21 downregulation, we now show that in colon tumors of *ACVR2A* KO mice pAkt activation is diminished and p21 levels are restored. Activation of PI3K is a potential mechanism of resistance to various cancer therapies [24, 26, 40] and when constitutively activated, therapeutic targeting of PI3K may be beneficial. In breast cancer cells, for instance, Akt may decrease p21 expression [41] enhancing to oncogenic behavior [42], which is consistent with our data regarding activin/PI3K/p21 downregulation.

p21 is a member of the Cip and Kip family of Cdk inhibitors, which includes p21, p27, and p57 [43]. These inhibit the kinase activity of broad, but not identical, classes of Cdk-cyclin complexes through their N-terminal homologous sequences. p21 arrests cell cycle progression primarily through the inhibition of Cdk2 activity, though it can also mediate p53-dependent G1 growth arrest. Earlier studies support the view that p21 suppresses tumors by promoting cell cycle arrest in response to various stimuli [44, 45]. While p21 regulation is often compromised in human cancers, its continuous expression, depending on the cellular context, suggests that it can act as either a tumor suppressor or an oncogene [43]. Deletion of p21 enhanced the rate of Ras- or c-Myc-induced tumorigenesis, and was associated with gene expression profiles and immunohistochemical features of EMT [29], consistent with our finding of enhanced migration with activin-induced p21 downregulation. Similarly, the loss of p21 enhanced the baseline total migration as well as activin-induced migration in SMAD4 intact cells, [9]. TGFβ induced p21 upregulation is decreasing over time and associated EMT.

As EMT is a central process in normal development, reactivation in cancer is regarded as dedifferentiation. It is typically characterized by the loss of cell-cell adhesion and apical-basal polarity, and may be induced by many different signaling pathways including TGFβ [20]. EMT manifests with repression of E-Cadherin often via Snail genes, and the development of a fibroblast-like motile phenotype [28]. Both SMAD-dependent and SMAD-independent signaling originating from TGFβ receptors have been implicated [3]. Context-dependent enhancement of EMT by additional mechanisms can include PI3K activation [46], as well as PDGF, EGF, and VEGF [20]. The molecular switch of TGFβ from tumor suppressive to oncogenic is also likely context-dependent, but remains poorly understood. We have previously reported a switch similar to that of TGFβ for activin signaling [9] and now show data implicating activin in EMT as well. TGFβ-induced EMT has been studied in detail but not much is known about the impact of activin signaling. There are some data that suggest that activin does not lead to EMT [18] or has a low impact on EMT [17], but experimental approaches were varied. Ansieau et al. report that Twist inhibits p21 leading to EMT and inhibition of oncogene-induced senescence [47] and our data suggests that TGFβ induced upregulation of p21 is decreasing over time in conjunction with increased EMT. Data from Barrallo-Gimeno et al. suggest long term exposure to TGFβ favors EMT over growth suppression [48], corresponding to our observations. Similarly, we show that while TGFβ increases p21 expression acutely [9], long term exposure (72 h and one week respectively) in colon cancer cells leads to downregulation by activin or loss of upregulation by TGFβ of p21 and subsequent induction of cell migration and EMT. We have previously reported that the downregulation of p21 leads to an increase of migration [9]. Our current data suggests a correlation between the downregulation of p21 and an increase in EMT. Small molecule inhibitors directed at growth factor receptors (such as EGFR) may interfere with EMT, although independent of primary receptor expression [20]. To forestall the development of autocrine loops, neutralizing antibodies against TGFβ ligand as well as combination therapy directed against EGFR and PI3K are being assessed [20].

## Conclusion

Understanding the complex molecular networks operating in cancer EMT is of utmost importance in the development of much needed biomarkers for risk and treatment stratification and for developing targeted combination therapies in advanced colon cancer. TGFβ and activin are prometastatic ligands in colon cancer which regulate EMT. These signaling molecules use identical upstream signaling that diverge at p21. Here, we show that activin and TGFβ actions in colon cancer are complex and involve distinct downstream mitogenic signaling. We further suggest that tumoral nuclear p21 may predict intact growth suppressive TGFβ signaling and may need to be assessed before planning trials with inhibition of TGFβ family pathways.

## Material and methods

### Colon cancer cell lines

SW480 (ATCC, Manassas, VA, USA) were maintained in DMEM, and FET cells (gift from Michael Brattain, University of Nebraska, Omaha, NE, USA) were maintained in DMEM/F12 50:50 (both Corning, Corning, NY, USA) supplemented with 10 % fetal bovine serum and penicillin (100 U/ml)/streptomycin (100 μg/ml) (Invitrogen, Carlsbad, CA, USA). Cells were grown at 37 °C in a humidified incubator with 5 % CO_2_. All cells were serum starved for 24 h prior to treatment to approximate cell cycle synchronization. Cells were validated by 9 STR (short tandem repeat) profiling using CellCheck 9 Plus and tested for mycoplasma (both IDEXX, Columbia, MO, USA).

### Reagents and antibodies

Activin A was reconstituted in PBS; TGFβ1 in 4 mM HCl according to the manufacturer’s instruction (both R&D, Minneapolis, MN, USA). Final concentrations used were 25 ng/ml and 10 ng/ml, respectively, as previously described [31, 49–51]. For inhibition of PI3K, we used LY294002 and for inhibition of MEK1/2, U0126 (both Cell Signaling Technology, Danvers, MA, USA). For immunoprecipitation and Western blotting, we used antibodies against ACVR1B (Santa Cruz Biotechnology, Santa Cruz, CA, USA), ACVR2A (customized by Yenzym, San Francisco, CA, USA), p85 (# 4292), TGFBR1 (# 3712) or TGFBR2 (# 3713, all Cell Signaling), p21 (# sc-65595, Santa Cruz), pan-Akt (# 8805, Abcam, Cambridge, MA, USA), GAPDH (# sc-47724, Santa Cruz), E-Cadherin (# 3195), vimentin (# 3390), pAkt Ser473 (# 4060) and pAkt Thr308 (# 13842, all Cell Signaling). For immunohistochemical analyses, we used p21 (# sc-817, Santa Cruz) ACVR2A (# ab10595), TGFBR2 (# ab78419), pAkt Ser473 (# ab81283, abcam), and pERK1/2 (# ab50011, all Abcam).

### Western blotting

Cells were lysed using CHAPS lysis buffer (containing 20 mM Bicine pH 7.6 and 0.6 % Chaps) with added protease and phosphatase inhibitors. Western blots were performed as previously described [9].

### siRNA and transfection

siRNA for SMAD4 (Ambion and Santa Cruz) and Akt1/2 (Santa Cruz) were transfected at a final concentration of 10 nM via electroporation using the AMAXA Nucleofector (Lonza, Basel, Switzerland) in 6-well plates at a density of 1×10^6^ per well according to the manufacturer’s instructions. Transfection efficiency was confirmed using the pmaxGFP™ Control Vector. Forty-eight hours post transfection, colon cancer cells were lysed for protein extraction.

### Migration assay

Migration assays were performed as previously described [31]. Briefly, transwell 12 well plates (8 μm pores, Corning, NY, USA) with fibronectin (Sigma, St. Louis, MO, USA) were seeded with 5 × 10^5^ colon cancer cells per well. Cells were then allowed to migrate for 6 h, stained, and imaged. Images from 5 microscopic fields at the center of each well were counted.

### Patient samples

110 colon cancer and adjacent normal tissue stains (fixed in formalin and embedded in paraffin) were obtained from Northwestern Memorial Hospital as de-identified, archived tissue samples under IRB approval. Additional File 4: Table S1 contains individual patient information.  Gender, age, tumor stage and expression of p21, TGFBR2 and ACVR2 is listed for each patient analyzed.

### Immunohistochemistry

Slides containing primary colon cancer and normal tissues were processed as previously described [52] and stained for AVCR2, TGFBR2, p21, pERK, and pAkt using the Catalyzed Signal Amplification System (CSA) by DAKO (Carpinteria, CA, USA). ACVR2A, TGFBR2, pERK, and pAkt staining was grouped into negative (no or weak signal) and positive (moderate or strong signal) status. The percentage of p21 positive nuclei in each cancer sample was assessed. p21 staining was grouped into nuclear (>50 % nuclei positive) or loss of nuclear (<50 % of nuclei positive). Slides were scored blindly by two investigators.

### Immunoprecipitation

1 mg of total protein lysate from various treatments were incubated with 1 μg of ACVR1B or ACVR2A antibody (Yenzym); ligand specificity was confirmed with TGFBR1 or TGFBR2 (Cell Signaling) overnight at 4 °C. Protein A beads (Invitrogen, Carlsbad, CA, USA) were added for 6 h. After denaturation with sample buffer, equivalent protein was fractionated on 4-20 % gradient gels (Biorad, Hercules, CA, USA), transferred to membranes and blotted with antibodies against ACVR1B antibody (Santa Cruz), and p85 (Cell Signaling).

### *ACVR2A* KO *in vivo mouse tumor model*

*ACVR2A* KO and *ACVR2A* wild type (wt) mice were treated with 14 mg/kg Azoxymethane (AOM) intraperitoneal infusion. After 5 days, mice were treated with 3 cycles of 2.5 % dextran sulfate sodium (DSS). Every cycle contained 5 days of DSS and 15 days of water. After day 100, the mice were sacrificed. Tumor and normal tissues were collected and lysed in RIPA buffer (1 % NP-40, 0.1 % SDS, 50 mM Tris–HCl pH 7.4, 150 mM NaCl, 0.5 % Sodium Deoxycholate, 1 mM EDTA) prior to use in Western blot analysis.

### Isoelectric focusing immunoassay

Detection of ERK1/2 phosphoisoforms after TGFβ and activin treatment was achieved using an automated capillary based isoelectric focusing immunoassay system (NanoPro1000 assay, ProteinSimple, Santa Clara, CA, USA). Protein isolation, detection, and quantification were done as per the manufacturer’s instructions. In brief, FET colon cancer cells were lysed in CHAPS buffer and 0.2 μg/ml protein was plated in a 384 well plate. For the primary antibody, we used pERK1/2 and ERK1/2 (Cell Signaling) diluted at 1:100. Luminescence and fluorescence images were collected using a charge-couple device (CCD) camera. Peak integration and isoelectric point (pI) marker calibration (for peak alignment) were performed using Compass, version 1.3.7, software (Protein Simple). The difference of isoform expression after TGFβ and activin treatment was compared to the vehicle control.

### Statistical analysis

Differences between groups were determined using the Student’s *t*-test. Probability values less than 0.05 were considered significant. Biological replicates from 3–5 experiments represent the data shown. For associations of IHC staining patterns, we performed Fisher’s exact test calculations.

## Additional files

Additional file 1: Table S2. Correlation of signaling pathway expression in primary colon cancer slides.(DOCX 73 kb) Additional file 2: Figure S1. ACVR2 interacts with p85 after activin treatment. FET cells were used for co-immunoprecipitation with ACVR2, TGFBR1 or TGFBR2 and Western blotted with p85.  There is an induction of ACVR2/p85 association following activin treatment, however, the increase in ACVR1/p85 association was more pronounced.  Neither TGFBR1 nor TGFBR2 directly associated with p85 following ligand stimulation. (EPS 3051 kb) Additional file 3: Figure S2. Activin-induced migration is reglated via PI3K and TGFbeta induced migration via MEK/ERK, both independent of SMAD4. Migration assays with FET cell lines were performed after 6 hours of ligand incubation and cells were compared by counting nuclei, stained blue with DAPI. (EPS 5130 kb) Additional file 4: Table S1. Characteristics of colon cancer patient cohort randomly selected from Northwestern University for p21, TGFBR2, ACVR2, pERK, and pAkt staining. Ten patients did not have stage information available (X). (DOC 170 kb)

## Abbreviations

ACVR: Activin receptor
TGFBR: TGFβ receptor
EMT: Epithelial mesenchymal transition
PI3K: Phosphatidylinositol-3’-kinase
