# Supplementary material for: Activin and TGFβ use diverging mitogenic signaling in advanced colon cancer
Source: Mol Cancer. 2015 Oct 24;14:182. doi: 10.1186/s12943-015-0456-4 (PMC4619565; doi:10.1186/s12943-015-0456-4)
Supplement: Additional file 1: Table S2. — Correlation of signaling pathway expression in primary colon cancer slides.(DOCX 73 kb) [file 12943_2015_456_MOESM1_ESM.docx]

**Supplementary table S2:** Correlation of signaling pathway expression in primary colon cancer slides.

A) loss of p21 and ARVC2+ are correlated with a p<0.0001 (Fisher’s exact test)

|  | ACVR2+ | ACVR2- | total |
| --- | --- | --- | --- |
| p21 - | 46 | 16 | 62 |
| p21 nuclear | 15 | 33 | 48 |
| total | 61 | 49 | 110 |

B) nuclear p21 and TGFBR2+ are correlated with a p<0.0001 (Fisher’s exact test)

|  | TGFBR2+ | TGFBR2- | total |
| --- | --- | --- | --- |
| p21 - | 19 | 42 | **61** |
| p21 nuclear | 38 | 11 | **49** |
| total | **57** | **53** | **110** |

C) pERK and ACVR2+ are not correlated (Fisher’s exact test)

|  | ACVR2+ | ACVR2- | total |
| --- | --- | --- | --- |
| pERK - | 27 | 20 | 47 |
| pERK + | 35 | 28 | 63 |
| total | 62 | 48 | 110 |

D) pAkt and TGFBR2+ are not correlated (Fisher’s exact test)

|  | TGFBR2+ | TGFBR2- | total |
| --- | --- | --- | --- |
| pAkt - | 26 | 24 | 50 |
| pAkt + | 31 | 29 | 60 |
| total | 57 | 53 | 110 |
