# Supplementary material for: Activin and TGFβ use diverging mitogenic signaling in advanced colon cancer
Source: Mol Cancer. 2015 Oct 24;14:182. doi: 10.1186/s12943-015-0456-4 (PMC4619565; doi:10.1186/s12943-015-0456-4)
Supplement: Additional file 4: Table S1. — Characteristics of colon cancer patient cohort randomly selected from Northwestern University for p21, TGFBR2, ACVR2, pERK, and pAkt staining. Ten patients did not have stage information available (X). (DOC 170 kb) [file 12943_2015_456_MOESM4_ESM.doc]

**Supplementary Table S1:** Characteristics of colon cancer patient cohort randomly selected from Northwestern University for p21, TGFBR2, ACVR2, pERK, and pAkt staining. Ten patients did not have stage information available (X).

| **Patient#** | **Gender** | **Age** | **Stage** | **p21** | **TGFBR2** | **ACVR2** |
| --- | --- | --- | --- | --- | --- | --- |
| 1 | M | 36 | IV | - | + | + |
| 2 | F | 68 | I | + | + | + |
| 3 | F | 63 | IV | - | - | + |
| 4 | M | 78 | III | - | - | + |
| 5 | M | 50 | III | - | - | - |
| 6 | F | 43 | II | + | - | - |
| 7 | M | 58 | III | - | - | + |
| 8 | F | 84 | III | - | + | - |
| 9 | F | 44 | III | + | - | - |
| 10 | M | 84 | IV | + | + | + |
| 11 | F | 64 | III | + | - | - |
| 12 | F | 83 | III | + | - | - |
| 13 | M | 68 | III | + | + | - |
| 14 | F | 65 | III | - | - | + |
| 15 | M | 76 | III | - | - | - |
| 16 | M | 82 | II | - | - | - |
| 17 | F | 52 | III | - | - | + |
| 18 | M | 54 | II | - | - | - |
| 19 | M | 50 | II | - | - | + |
| 20 | F | 70 | II | + | + | - |
| 21 | F | 55 | II | - | - | + |
| 22 | F | 84 | III | - | - | + |
| 23 | M | 81 | II | - | - | + |
| 24 | M | 56 | III | + | + | - |
| 25 | M | 49 | III | + | + | - |
| 26 | M | 55 | III | + | + | - |
| 27 | F | 64 | X | - | - | + |
| 28 | M | 28 | III | + | + | - |
| 29 | M | 42 | II | - | + | + |
| 30 | M | 84 | III | + | + | - |
| 31 | M | 70 | IV | - | - | + |
| 32 | M | 52 | IV | + | - | + |
| 33 | F | 64 | III | - | + | + |
| 34 | F | 75 | X | + | + | + |
| 35 | M | 76 | X | + | + | - |
| 36 | M | 72 | III | - | - | + |
| 37 | F | 81 | II | - | - | - |
| 38 | M | 79 | III | + | + | + |
| 39 | F | 73 | II | + | + | - |
| 40 | F | 81 | III | + | + | - |
| 41 | F | 70 | III | - | + | - |
| 42 | M | 96 | III | + | + | - |
| 43 | F | 69 | III | + | + | - |
| 44 | M | 72 | III | - | - | + |
| 45 | F | 37 | III | + | + | + |
| 46 | F | 36 | III | + | + | - |
| 47 | F | 83 | III | - | - | + |
| 48 | F | 60 | III | + | - | - |
| 49 | F | 69 | X | - | - | + |
| 50 | F | 77 | III | - | - | - |
| 51 | M | 60 | X | - | - | + |
| 52 | F | 56 | III | - | - | + |
| 53 | M | 75 | II | + | + | - |
| 54 | M | 68 | I | + | + | - |
| 55 | F | 64 | III | - | + | + |
| 56 | F | 82 | III | - | + | - |
| 57 | F | 73 | III | - | - | + |
| 58 | M | 56 | I | + | - | + |
| 59 | M | 54 | III | + | + | + |
| 60 | M | 75 | II | - | - | - |
| 61 | F | 56 | III | + | - | - |
| 62 | F | 64 | III | - | - | + |
| 63 | F | 82 | III | - | - | + |
| 64 | M | 54 | III | + | + | - |
| 65 | F | 77 | III | - | + | + |
| 66 | M | 60 | X | - | - | - |
| 67 | M | 56 | I | + | + | + |
| 68 | F | 73 | III | - | + | + |
| 69 | M | 68 | I | + | + | - |
| 70 | F | 70 | II | - | + | + |
| 71 | F | 71 | III | + | + | - |
| 72 | F | 71 | IV | - | + | + |
| 73 | F | 40 | III | + | + | - |
| 74 | F | 55 | III | - | + | + |
| 75 | F | 55 | II | - | + | + |
| 76 | M | 78 | II | + | + | + |
| 77 | F | 34 | IV | - | - | - |
| 78 | M | 79 | III | + | + | - |
| 79 | M | 80 | I | - | - | - |
| 80 | F | 50 | IV | + | + | - |
| 81 | M | 73 | III | + | + | - |
| 82 | M | 82 | X | - | - | + |
| 83 | M | 49 | X | + | - | - |
| 84 | M | 49 | IV | - | - | + |
| 85 | F | 62 | III | - | - | + |
| 86 | M | 47 | III | - | + | + |
| 87 | M | 80 | III | + | + | + |
| 88 | F | 74 | III | - | - | - |
| 89 | M | 48 | III | - | + | + |
| 90 | M | 63 | X | - | - | + |
| 91 | F | 70 | I | - | + | + |
| 92 | F | 72 | IV | - | - | + |
| 93 | F | 52 | IV | + | + | - |
| 94 | F | 70 | III | + | + | + |
| 95 | F | 57 | II | + | - | + |
| 96 | M | 37 | II | - | - | + |
| 97 | M | 41 | X | + | + | - |
| 98 | M | 65 | III | - | - | - |
| 99 | M | 65 | IV | + | + | + |
| 100 | M | 45 | IV | - | - | + |
| 101 | F | 85 | I | - | - | + |
| 102 | F | 72 | I | + | - | + |
| 103 | M | 37 | I | + | + | + |
| 104 | F | 58 | IV | + | + | - |
| 105 | M | 82 | IV | - | - | + |
| 106 | M | 78 | III | + | + | - |
| 107 | M | 63 | III | - | + | + |
| 108 | M | 69 | III | - | + | + |
| 109 | M | 64 | III | - | - | + |
| 110 | F | 84 | IV | - | + | + |
